# Traditionally removed mandibular central incisors and oral health-related quality of life: a cross-sectional study among adolescents in Maasai populated areas, Northern Tanzania

**DOI:** 10.1186/s12903-024-04060-9

**Published:** 2024-03-11

**Authors:** Lutango D Simangwa, Anne N Åstrøm, Anders Johansson, Irene K Minja, Ann-Katrin Johansson

**Affiliations:** 1 Department of Dentistry – Oral Health Services, Katima Mulilo State Hospital, Katima Mulilo, Namibia; 2 Department of Clinical Dentistry – Community Dentistry, Faculty of Medicine, University of Bergen, Bergen, Norway; 3 Department of Clinical Dentistry - Prosthodontics, Faculty of Medicine, University of Bergen, Bergen, Norway; 4 Department of Restorative Dentistry, School of Dentistry, Muhimbili University of Health and Allied Sciences, Dar Es Salaam, Tanzania; 5 Department of Clinical Dentistry - Cariology, Faculty of Medicine, University of Bergen, Bergen, Norway

**Keywords:** Adolescents, Anterior teeth, Maasai population areas, Mandibular central incisors, Oral impact on daily performances, Traditional tooth extraction

## Abstract

**Background:**

The traditional removal of mandibular anterior teeth has been existing for many years in the Sub-Saharan African countries. This study aimed to assess the prevalence and sociodemographic distribution of traditionally removed mandibular central incisors (TRMCI) and its association with oral impact on daily performance (OIDP) among adolescents in Maasai populated areas in the Northern part of Tanzania.

**Methods:**

Using a two-stage cluster sample design, with schools as the primary sampling unit, 23 out of 66 eligible rural schools were randomly selected. From each selected school, one class, expected to contain adolescents aged 12–14 years, was identified. The students from these selected classes were invited to participate in the study. A total of 989 adolescents were invited and 906 (91.6%) accepted to participate and completed both an interview and a clinical oral examination.

**Results:**

Mean age was 13.4 years (12–17 years, SD 1.2) and 43.9% were males (*n* = 398). The participants from Longido district amounted to 47.1%. The Maasai group constituted 79.6% of the study participants. The frequency of the participants missing at least one mandibular central incisor were 18.5%. Multivariable logistic regression revealed that adolescents from Longido district were more likely to report at least one TRMCI (OR = 2.5, 95% CI 1.4–3.3). Adolescents from non-Maasai group were less likely to have atleast one TRMCI compared to adolescents from Maasai ethnic group (OR = 0.02, 95% CI 0.002–0.15). Adolescents with at least one TRMCI were more likely to report impacts on OIDP (OR = 3.3, 95% CI 1.9–5.7) than those without TRMCI. Independent of the TRMCI status, adolescents from Longido district were less likely than their counterparts to report oral impacts (OR = 0.4, 95% CI 0.2–0.6). Similarly, adolescents from non-Masaai group were more likely than their counterparts to report oral impacts (OR = 2.2, 95% CI 1.4–3.5).

**Conclusion:**

TRMCI is common among adolescents in the Maasai populated areas in the Northern part of Tanzania and strongly associated with the district of residence and Maasai ethnicity and has a negative impact on oral health related quality of life. There is a need for oral health education in the rural Maasai communities in Tanzania to increase awareness of the negative consequences of this practice.

## Background

The traditional removal of mandibular/maxillary anterior teeth is a form of dental mutilation. The practice has been existing for more than 1500 years ago and was reported mainly from Sub-Saharan African countries [1, 2]. Studies from for example Sudan, Uganda Tanzania and Kenya, but also from Angola, the Democratic Republic of Congo and Namibia have been reporting on the presence of this traditional removal of teeth [1, 3–6]. Besides this, the practice has been reported among immigrants in developed countries and indigenous people of under-developed countries [7, 8].

The rationale behind this traditional practice varies but includes for example cultural and ethnic identification as well as a sign of respect for the tribal chieftains and as a mark of high status and beauty. Historically, it is believed that the practice started in Africa, when there was high incidence rates of tetanus more than 1500 years ago [2]. One symptom of the disease was a lockjaw which made both eating and breathing difficult. To ease this problem, it is known that the Maasai people in Kenya removed the anterior incisors. In addition, they also performed another ritual where their children’s mandibular canines were extracted. The reason for this was that the Maasai people had noticed that their children in connection to teething suffered from both diarrhea and febrile diseases and that their bovine calves, who did not have any canines, did not. Thus, by (also) removing the canines the Maasai people wanted their children to avoid teething complications [9, 10]. Studies from both Sudan and South Africa have reported on the removal of mandibular permanent incisors in order to facilitate oral sex. In South Africa, this practice has been reported mostly among families with low socio-economic status [8, 11].

The above-mentioned types of dental mutilations are often performed by traditional healers under relatively poor hygienic conditions and without anesthesia [12]. Complications which can arise from this procedure can be divided into immediate complications and long term complications. The most commonly reported immediate complications are pain, excessive bleeding, anemia, and various types of infections, including tetanus, and in some cases, the procedure might end up in death [13]. Long term complications include malnutrition, unwanted side effects on the occlusion may occur, for example, elongation (overeruption) of teeth as a result of the missing antagonists. This may have an adverse impact on the function of the masticatory system including eating, smiling, and speaking as well as on other social interactions, which in turn, may lead to psychological consequences [14–16].

The prevalence of missing anterior teeth due to traditional removal varies not only between countries but also within the same country as well as among ethnic groups. In Kenya in 2001, it was reported that 12.3% of all missing teeth among Kenyans (6–85 years) were a result of traditional removal [17, 18]. In another study conducted in 2016, it was reported that 61% of Kenyan adolescents (14–18 years) had various forms of oral mutilations. 95% of these mutilations was a result of bilateral removal of the mandibular central incisors [17, 18].

In Tanzania, information on traditional tooth removal is rare. In one study from 1991 covering different areas of Tanzania, it was found that 8% of the participants (12–19 years), had mutilated permanent teeth as a result of traditional removal [19]. In another study from South East Tanzania in 2007 focusing on adults from the Makonde people, the prevalence of mandibular anterior tooth loss due to dental mutilation was estimated to 22% [20].

Tooth loss adversely affects the oral health related quality of life (OHRQoL) by disturbing, for example, chewing function, facial attractiveness, and social interaction [21, 22]. OHRQoL refers to how oral diseases affect an individual’s ability to function in terms of chewing, speaking, smiling, psychosocial status, pain, and discomfort. Poor oral health may cause various complications and costs to both individuals and healthcare providers and also to society at large [21]. This may lead to poor self-esteem for individuals and low economic productivity [23, 24]. Several inventories have been developed to assess OHRQoL among children and adolescents, for example, the early childhood impact scale, Child Perceptions Questionnaire (CPQ11-14), parental perception questionnaire, family impact scale, and the Child Oral Impact on Daily Performance (Child-OIDP) [25–29]. Only a few OHRQoL inventories have been utilized in a Sub-Saharan African context.

As there is a paucity of recent research on traditionally removed mandibular central incisors (TRMCI) in Tanzania, there is a need to further explore this practice, particularly among socially disadvantaged minority groups such as the Maasai adolescents. In Tanzania, the Maasais are considered socially disadvantaged because they live in remote areas where social services are limited, they have poor knowledge of Swahili, the national language. These factors reduce chances for health services provision and education achievement [30]. Therefore, this study aimed to assess the prevalence and sociodemographic and clinical distribution of TRMCI and the association between TRMCI and OIDP among adolescents attending primary schools in Maasai populated areas in Arusha region, Northern part of Tanzania.

## Methods

### Sampling technique and sample size

A cross-sectional study, focusing primary school adolescents living in Maasai populated areas of Monduli and Longido districts, Arusha region in the Northern part of Tanzania, was performed between June and November 2016. The sample size estimated was 845 study participants based on the assumption that the prevalence of TRMCI among adolescents was 50%, with a margin error of 5% and confidence intervals of 95%. Furthermore, the sample size was multiplied by 2 to account for the design effect (D), and increased by 10% to account for contingencies.

A list of all primary schools including both public and private schools (total of 100 schools) was obtained from both districts. All urban and private primary schools from the list were excluded and the remaining 66 rural public primary schools, were included in the sampling frame. Using a two-stage cluster sample design with schools as the primary sampling unit, 23 out of 66 eligible public rural schools were selected. In every randomly selected school, one class expected to contain adolescents aged 12–14 years (6th graders) was purposely identified. From the identified classes, all adolescents in the appropriate age range were invited to participate in the study. Details for the sampling procedure and other methods used in this paper has been described elsewhere [45].

### Interview

A face-to-face interview with each participant was performed at the school area by trained medical nurses native to the study area and fluent in both Swahili, the national language, and Maa, the Maasai language. A questionnaire (interview schedule) with closed- and open-ended questions was used when the participants were interviewed [45]. This schedule was constructed in English, translated into Kiswahili, and back-translated to English independently by professional language translators in Tanzania. Prior to this study, a pilot study comprising 50 participants (12–14 years), was carried out to test the questionnaire. Where appropriate, the schedule was adjusted in terms of wording and meaning, as well as the appropriateness of its format.

Socio-demographic characteristics were assessed in terms of the district of residence, sex, age, ethnicity, wealth index, region of birth, mother’s education, father’s education, house ownership and the number of children in a family. The wealth index was assessed by asking about the presence/absence of durable household assets indicative of family wealth (i.e. radio, television, refrigerator, mobile telephone, cupboard, bicycle, and motorcycle) and was recorded as *(yes) “available and in working condition”* or *(no) “not available and/or not in working condition”.* Region of birth was assessed by asking *“Have you lived in the same area of residency since birth”?* The response was either *“yes”* or *“no”.* House ownership was assessed by asking *“Who owns the house your family is living in at the moment?* The response- alternatives were *“owned by your family*”, *“rented house”* or “*I don’t know”*. Number of children in a family was assessed by the open-ended question *“How many children are there in your family?*”

Temporomandibular disorders (TMD) were assessed by asking two validated epidemiological questions: “*Do you have pain in your temple, face, jaw or jaw joint once a week or more?”* and *“Does it hurt once a week or more when you open your mouth or chew?”* The response was either “*yes”* or *“no”* and a positive response to one or both of the two questions was considered affirmative to TMD diagnosis [46].

The method used to measure oral health related quality of life (OHRQoL) has been described previously [47]. OHRQoL was measured using a validated Kiswahili version of the eight item Child Oral Impacts on Daily Performance (OIDP) inventory [48]. The Child-OIDP frequency index referred to difficulty carrying out eight daily life activities *“During the past 3 months, how often have problems with your mouth or teeth caused you any difficulty with; eating and enjoying food, speaking and pronouncing clearly, cleaning teeth, sleeping and relaxing, smiling and laughing, emotional status, socialization and contact with people.* The responses were (0) *never, (1) once or more a month, (2) once or more a week and (3) every day/nearly every day.* Each item was dichotomized as 0 not affected (comprising original response 0) and 1 affected (comprising original responses 1, 2, and 3). A Child-OIDP simple count (SC) score (range 0–8) was constructed by summing the dichotomized frequency items and subsequently dichotomize into 0 (original SC score 0, no impacts) and 1 (original SC 1–8, at least one impact). In its original form, OIDP scores are calculated by multiplying frequency and severity scores of daily performances. However, evidence suggests the use of either frequency or severity scores for reasons of simplicity and efficiency [49].

### Clinical oral examination

All clinical oral examinations were performed by one investigator (LS.) in natural daylight and under field conditions. The study participant was examined using disposable mouth mirrors and a sickle probe while sitting on a chair. Gingival bleeding, dental caries, dental erosion, and tooth wear were recorded using Gingival Bleeding Index (GBI), criteria specified by WHO 2013 (DMFT), Johansson et al. 1996 and Carlsson et al. 1985 (dental erosion, dental wear), respectively [50–53]. Mandibular central incisors were recorded as present or absent during the examination. Those participants who missed at least one mandibular central incisor were further asked two questions during the clinical examination by the examiner. (1) Who removed your tooth/teeth? (2) What was the reason for removing your tooth/teeth?

### Statistical analysis

The Statistical Package for Social Sciences (SPSS) for Personal Computer (PC) version 23 (IBM corporation, Armonk, NY, USA) and STATA 14.2 (Stata Corporation, Lakeway drive college station, Texas, USA) were used for data analyses. Descriptive statistics were carried out followed by bivariate analysis using cross-tabulations and Pearson’s chi-square statistical test. The percentage agreement and Cohen’s Kappa were used to examine the intra-examiner concordance. Multiple variable logistic regression analyses were performed using odds ratio (OR) and 95% confidence intervals (CI). Sociodemographic factors and oral diseases/conditions, significantly associated with TRMCI in unadjusted analysis were included in the multiple logistic regression model, setting the level for statistical significance to *p* < 0.05. In the final model, p-value < 0.05 was considered significant. Multiple variable logistic regression was also used to estimate the adjusted association of TRMCI with OIDP, controlling for socio-demographic and clinical oral health characteristics that were associated with TRMCI and OIDP in unadjusted analyses. Analyses were adjusted for the primary sampling unit, school, using a survey design.

## Results

### Sample characteristics

In the study a total of 989 grade 6 primary school adolescents from 23 rural schools were invited to participate and 930 (of those) accepted to participate. The response rate was 91.6%. Due to too high or low age, 24 (2.6%) of the adolescents were excluded during analysis. Finally, the study included 906 participants (12–17 years, mean age of 13.4 years (SD 1.2) of which 398 (43.9%) were males. Among the study participants, 47.1% were from Longido district, and 52.9% were from Monduli district. The Maasai group constituted 79.6% of the total number of participants. Details of the sociodemographic distribution of the total study group are shown in Table 1.

**Table 1:** Socio-demographic distribution of the study participants, *n* = 906

Variable	Categories	Participants % (n)
District of residence	Monduli Longido	52.9 (479) 47.1 (427)
Sex	Male Female	43.9 (398) 56.1 (508)
Age group	12–14 years 15–17 years	85.8 (777) 12.5 (113)
Ethnic group	Maasai Non Maasai	79.6 (721) 20.4 (185)
Wealth index	Poorest Least poor	48.3 (438) 50.7 (459)
Region of Birth	Arusha Others	94.8 (859) 5.2 (47)
Mother’s education	Low (≤ primary school) High (≥ secondary school)	95.0 (861) 5.0 (45)
Father’s education	Low (≤ primary school) High (≥ secondary school)	92.1 (834) 7.9 (72)
House ownership	Yes No	94.8 (859) 5.2 (47)
Number of children	1–5 children 6–14 children	48.9 (443) 51.1 (463)

### Reliability

To assess the intra-examiner concordance (LS), duplicate clinical examinations were carried out with 93 randomly selected participants three weeks apart. Analysis performed on duplicate examination records revealed Kappa value of 0.99, 0.87, and 0.69 for at least missing one mandibular central incisor, TF-index, and dental erosion respectively.

### Frequency of TRMCI and its sociodemographic and clinical distribution

**Fig. 1:**
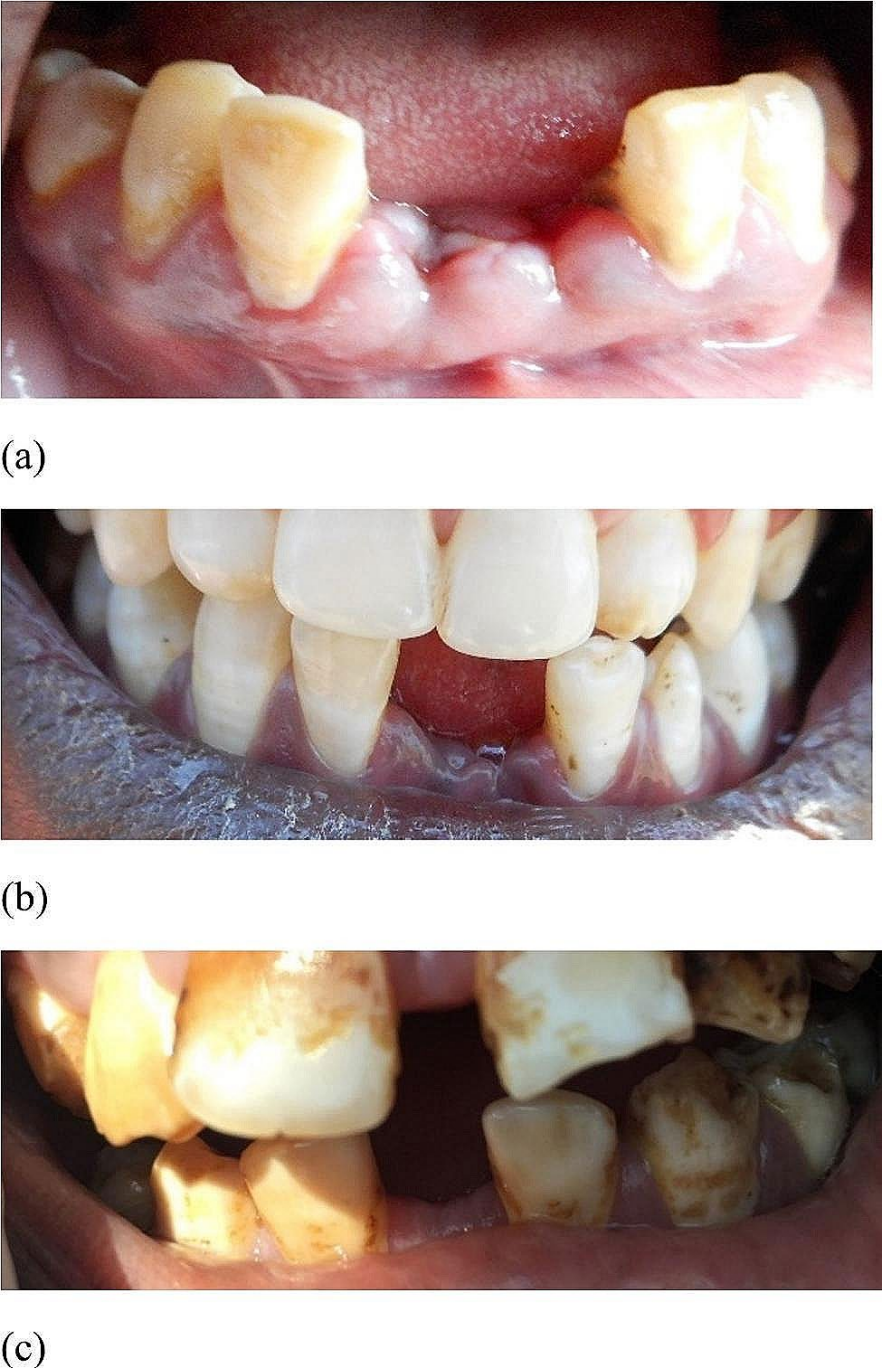
Three different Maasai adolescents from this study with missing mandibular central incisors due to traditional removal of teeth (a-c). (**a**) The central mandibular incisors were removed three days before the clinical examination of this study. (**b**) Space reduction between mandibular lateral incisors has not occurred (**c**) Spaces between the lateral incisors is reduced after removal of mandibular central incisors

A total of 168/906 (18.5%) of the study participants missed at least one mandibular central incisor (31 and/or 41) and confirmed that this was the result of a traditional removal of these teeth (TRMCI). Figure 1 is showing photographs of a participant with central mandibular incisors removed due to traditional practice. Regarding the question “Who removed your tooth/teeth?”, all of the participants responded that they were removed by their respective elders, parents or grandparents. They further declared that the removal was not done in a hospital. Regarding the question “What was the reason for removing your tooth/teeth?*”*, all of the adolescents except one did not know why their tooth/teeth were extracted. The only study participant who knew the reason for removing their teeth said that “this is Maasai tribe, some Maasai adolescents remove their lower jaw central teeth for the ethnic identification”. There were only a small number of other missing teeth in the anterior region: tooth 13 (1.5%), tooth 12 (0.4%), tooth 11 (0.3%), tooth 21 (0.1%), tooth 22 (0.2%), tooth 23 (1.4%), tooth 33 (0.8%), tooth 32 (1.1%), tooth 42 (1.4%) and tooth 43 (1.2%).

TRMCI varied statistically significantly with sociodemographic characteristics in terms of district of residence, ethnicity, region of birth, mothers’ and fathers’ education and by dental erosion (Table 2). A total of 14.6% and 23.0% of adolescents from Monduli and Longido districts had at least one TRMCI. Whereas 23.2% of Maasai adolescents had at least one mandibular central incisor removed, this was the case for only 0.5% of non-Maasai adolescents. Also 19.4% of adolescents who were born within Arusha region had at least one TRMCI compared to those who were born outside Arusha region (2.1%). In addition, 19.4% of adolescents born from mothers with low level of education reported at least one TRMCI compared to those from mothers with high level of education (2.2%).

**Table 2:** Unadjusted regression analysis. Association between socio-demographic, clinical variables and TRMCI and association between socio-demographic, clinical variables and OHRQOL

Variable	TRMCI % (n)	p-value**	OIDP > 0 % (n)	p-value**
District of residence Monduli Longido	14.6 (70) 23.0 (98)	0.001	21.9 (105) 8.9 (38)	< 0.001
Sex Female Male	19.3 (98) 17.6 (70)	0.513	14.2 (72) 17.8 (71)	0.133
**Age** 12–14 years 15–17 years	17.6 (137) 23.0 (26)	0.167	15.2 (118) 22.1 (25)	0.061
**Ethnicity** Maasai Non-Maasai	23.2 (167) 0.5 (1)	< 0.001	14.6 (105) 20.5 (38)	0.047
**Wealth index** Poorest Least poor	19.9 (87) 17.4 (80)	0.349	11.9 (52) 19.4 (89)	0.002
**Region of birth** Arusha Others	19.4(167) 2.1 (1)	0.003	15.7 (135) 17.0 (8)	0.811
**Mother’s education** Low (≤ primary school) High (≥ secondary school)	19.4 (167) 2.2 (1)	0.004	15.4 (133) 22.2 (10)	0.224
**Father’s education** Low (≤ primary school) High (≥ secondary school)	19.8 (165) 4.2 (3)	0.001	15.2 (127) 22.2 (16)	0.118
**Number of children** 1–5 children 6–14 children	17.6 (78) 19.4 (90)	0.478	16.7 (130) 10.2 (13)	0.064
**Gingival bleeding** No Yes	16.6 (89) 21.3 (79)	0.076	14.8 (79) 17.3 (64)	0.313
**DMFT** DMFT = 0 DMFT > 0	18.6 (154) 17.5 (14)	0.802	13.7 (113) 37.5 (30)	< 0.001
**Dental fluorosis** TF score 0–4 TF score 5–9	19.5 (91) 17.5 (77)	0.432	11.4 (53) 20.5 (90)	< 0.001
**Dental erosion** Grade 0 Grade > 0	20.6 (130) 13.9 (38)	0.017	16.8 (106) 13.5 (37)	0.215
**Tooth wear** Grade 0 Grade > 0	16.8 (82) 20.6 (86)	0.137	14.9 (73) 16.8 (70)	0.445
**TMD pain** 2Q/TMD* = 0 2Q/TMD*> 0	17.9 (143) 23.4 (25)	0.172	12.3 (98) 42.1 (45)	< 0.001
**TRMCI** TRMCI = 0 TRMCI > 0			14.9 (110) 19.6 (33)	0.128
**OIDP** Impact No impact	23.1 (33) 17.7 (135)	0.129		

*2Q/TMD Two epidemiological questions regarding TMD pain

**Pearson’s Chi-square test

Similarly, OIDP varied statistically significantly by the district of residence whereby 8.9% of adolescents from Longido reported at least one OIDP compared to 21.9% of adolescents from Monduli district. In addition, oral impacts (OIDP > 0) was more frequently reported among the non-Maasai (20.5%) compared to Maasai adolescents (14.6%) (*p* < 0.05), among the least poor (19.4%) compared to the poorest (11.9%) and among adolescents with caries experience (37.5%) compared to their caries free counterparts. Totals of 14.9% versus 19.6% of adolescents with and without at least one TRMCI, respectively, reported at least one oral impact on daily performances. For details of other independent variables see Table 2.

Socio-demographic and clinical variables that were statistically significantly associated with TRMCI (p-value < 0.05) in unadjusted analysis (Table 2) were simultaneously included into a multiple variable logistic regression model. As depicted in Table 3, adolescents from Longido district were 2.5 times (OR = 2.5, CI 1.4–3.3) more likely to report at least one TRMCI compared to those from Monduli district. Adolescents from the non-Maasai ethnic group were less likely to report TRMCI as compared to their Maasai counterparts. Other socio-demographic and clinical covariates were not independently associated with TRMCI in the multiple variable analysis.

**Table 3:** Adjusted regression analysis. TRMCI regressed on socio-demographic features, and clinical findings. Adjusted odds ratios (OR) and 95% confidence intervals (CI)

Variable	TRMCI	
	OR (95%CI)	P-value
**District of residence** Monduli Longido	1 2.5 (1.7–3.5)	< 0.001
**Ethnicity** Maasai Non Maasai	1 0.02 (0.002–0.15)	< 0.001
**Region of birth** Others Arusha	1 3.0 (0.4–25.4)	0.30
**Mother’s education** Low (≤ primary school) High (≥ secondary school)	1 0.2 (0.02–1.3)	0.09
**Father’s education** Low (≤ primary school) High (≥ secondary school)	1 0.3 (0.1–1.2)	0.09
**Dental erosion** Grade 0 Grade > 0	1 0.7 (0.5–1.1)	0.08

Table 4 depicts the findings from multiple logistic regression analyses with TRMCI regressed on OIDP and adjusted for potentially confounding socio-demographic variables. Socio-demographic factors that were statistically significantly associated with both OIDP and TRMCI in unadjusted analysis were included in the multivariable analysis, whereas sex and age group were forced into the model in order to control confounding. Adolescents with TRMCI > 0 were 3.3 times more likely to report oral impacts (OR = 3.28, 95% CI 1.9–5.7) than those without TRMCI. Independent of the TRMCI status, adolescents from Longido district were less likely than their counterparts to report oral impacts (OR = 0.4, 95% CI 0.2–0.6). Adolescents from non- Masaai group were more likely than their counterparts to report oral impacts (OR = 2.2, 95% CI 1.4–3.5). A significant two-way interaction was observed between TRMCI and the district (OR = 0.2, 95% CI 0.07–0.6). The stratified analysis revealed that in Monduli, adolescents with TRMCI > 0 were 2.8 times more likely than their counterparts to report oral impacts, (OR = 2.8, 95% CI 1.7–4.9). In Longido, the ORs for oral impacts if having TRMCI > 0 was not statistically significant.

**Table 4:** OIDP regressed on TRMCI and adjusted for socio-demographic factors. Adjusted odds ratios (OR) and 95% confidence intervals (CI)

	OR 95% CI	p-value
**Traditionally removed mandibular central incisor (TRMCI)**		
TRMCI = 0	1	
TRMCI > 0	3.3 (1.9–5.7)	0.001
**Districts**		
Monduli	1	
Longido	0.4 (0.2–0.6)	0.001
**Sex**		
Male	1	0.049
Female	0.7 (0.5–0.9)	
**Age**		
12–14 years	1	0.674
15–17 year	1.1 (0.7–1.8)	
**Ethnicity**		
Maasai	1	0.001
Non- Maasai	2.2 (1.4–3.5)	

## Discussion

To our knowledge, this is the first study reporting on the prevalence of traditionally removed mandibular central incisors (TRMCI), the sociodemographic and clinical distribution and any impact on oral health-related quality of life among adolescents attending primary schools in Maasai populated areas in Arusha region in the Northern part of Tanzania. The practice of traditionally extracted central mandibular incisors was a common finding.

The prevalence of TRMCI (18.5%) reported in this study is higher than reported dental mutilation in an Ethiopian population (7%), similar to a Sudanese population (22%) but substantially lower that that found in Kenya (61%) [18, 31, 32]. Consequently, the practice of dental mutilation exhibits great regional differences even among the same ethnic groups. Missing of mandibular anterior teeth may occur due to for example trauma, tooth impaction or agenesia as well as genetics or infections [33–35]. Congenital aplasia and/or traumatic avulsions of mandibular incisors has been found to be rare, and a prevalence of 0.25–1.5% has been reported [36–38]. Considering the much higher prevalence of TRMCI found in this study, congenital/traumatic reasons can be excluded as a main cause.

Adolescents from Longido district were almost three times more likely to have missing mandibular central incisors compared to adolescents from Monduli district. The Longido district is located in the northern part of Tanzania, close to the border to Kenya, while Monduli is more south. Studies among Kenyan Maasai adolescents has, as mentioned earlier, reported a high prevalence of dental mutilations [18]. It is likely that the Maasai society from Longido district have more social and cultural interactions with the Maasais from Kenya than those living in the more southern located Monduli district. This might have influenced the higher prevalence of TRMCI in the Longido district. In analogy with TMRCI, the practices of tooth bud extraction (mainly un-erupted deciduous canines) which is another type of intraoral dental mutilation, has been found to be common in neighboring Uganda [39].

Findings from this study, revealed that low parental education level was strongly correlated with the TRMCI which is in agreement with previous reports from Somalia and Uganda [40, 41]. It is well known that children from parents with high education tend to be healthier than children from parents with low education level. In the Maasai community in Tanzania, education might have a direct impact on adolescent health as it increases the capacity to acquire knowledge and process various information regarding oral health and general health at large. Parents with higher educational attainment are more likely to have the knowledge, resources, and access to health care that are necessary for promoting optimal oral health for their adolescents [42].

In this study, the majority of adolescents with missing mandibular central incisors due to traditional tooth extraction were from the Maasai ethnic group and only 0.5% of the non-Maasai participants. However, the finding may be explained by the fact that the Maasai ethnic group in the study area to a higher extent maintain their traditional lifestyle. In this regard, the only participant who thought to know the reason said it is performed for “ethnic identification”. Most likely there will be some changes in the Maasai lifestyle in the future due to more interactions and influences from other ethnic groups and also due to the development of the society at large.

Although the prevalence of TRMCI was greater in Longido than Monduli, its impact on oral health related quality of life was only present in Monduli. It is also interesting that adolescents in this study who had their central mandibular teeth removed were about three times more likely to report at least one oral impact on daily performance than their counterparts without TRMCI. This finding is in contrast to a study of Brazilian adolescents where no association with tooth loss found with compromised oral quality of life [43]. OHRQoL is commonly found to adversely affected by tooth loss due to its effect on mastication of food and nutritional status [23]. According to previous reports, facial attractiveness has been shown to affect attitudes, actions and employment situations. Missing teeth markedly affect the individual appearance leading to a negative impression on prospective employers and less self-esteem for individuals [21, 23]. This could be one explanation for the correlation between TRMCI and OHRQoL in this study.

The negative consequences reported for TRMCI are manifold. Pain, blood loss and poor infection control may lead to septicemia/tetanus and also to transmission of blood-borne diseases such as HIV/AIDS. The practice may also lead to malocclusions and psychological and/or social embarrassment [16, 44]. Thus, the negative consequences are many and stresses the need for oral health education in these groups in order to prevent this practice especially among the Maasai ethnic group. The supposed benefits for the participants of this traditional practice were not investigated but objectively there seems to be none except for that it might contribute for giving an ethnic identification for the Maasais.

### Limitations of the study

In this study, the primary sampling units (schools) were randomly selected, thus increasing the likelihood that the present findings might be representative to the larger communities of adolescents living in Maasai populated areas. Individual adolescents were the units of analyses, however for the purpose of determining good precision of the estimates, data clustering was accounted for in statistical analyses. The research assistants we used for the interview were medical nurses and fluent in both Kiswahili and Maasai language (Maa) and this made the adolescents understand the questions during the interview. In addition, one examiner performed the clinical examinations of all children thus reducing any inter-examiner variability. However, sometimes using one examiner only may lead to the introduction of observer effect bias and confirmation bias. In observer effect bias, the observer may influence the participants of the study and in confirmation bias, the experimenter interprets the results incorrectly because of the tendency to look for information that conforms to their hypothesis and overlook information that argues against it. The background factors were based on self-report, thus posing a possibility that the child might have misunderstood the question, forgotten, or that the response might be influenced by social desirability. The cross-sectional method utilized in data collection makes it difficult to establish a causal relationship.

## Conclusions

The traditional extraction of mandibular central incisors is common among adolescents in the Maasai populated areas in the Northern part of Tanzania and strongly associated with the district of residence and Maasai ethnicity and had a negative impact in oral health related quality of life. Thus, there is a need for oral health education in the rural Maasai communities in Tanzania to increase awareness of the negative consequences of this practice.

## Data Availability

The datasets used and/or analysed during the current study are available from the corresponding author on request.

## Abbreviations

CI: Confidence Interval
CPQ: Child Perceptions Questionnaire
DMFT: Decayed Missing Filled Tooth
GBI: Gingival Bleeding Index
HIV/AIDS: Human Immunodeficiency Virus / Acquired Immunodeficiency Syndrome
OHRQoL: Oral Health Related Quality of Life
OIDP: Oral Impact on Daily Performance
OR: Odds Ratio
SC: Simple Count
SD: Standard deviation
SPSS: Statistical Package for Social Sciences
TMD: Temporomandibular Disorder
TRMCI: Traditionally Removed Mandibular Central Incisors
WHO: World Health Organization
